# Warm acclimation alters antioxidant defences but not metabolic capacities in the Antarctic fish, *Notothenia coriiceps*

**DOI:** 10.1093/conphys/coac054

**Published:** 2022-08-02

**Authors:** Kristin M O’Brien, Corey A Oldham, Jon Sarrimanolis, Autumn Fish, Luke Castellini, Jenna Vance, Hayley Lekanof, Elizabeth L Crockett

**Affiliations:** Institute of Arctic Biology, University of Alaska, Fairbanks, AK 99775 USA; Institute of Arctic Biology, University of Alaska, Fairbanks, AK 99775 USA; Institute of Arctic Biology, University of Alaska, Fairbanks, AK 99775 USA; Institute of Arctic Biology, University of Alaska, Fairbanks, AK 99775 USA; Institute of Arctic Biology, University of Alaska, Fairbanks, AK 99775 USA; Department of Biological Sciences, Ohio University, Athens, OH 45701 USA; Institute of Arctic Biology, University of Alaska, Fairbanks, AK 99775 USA; Department of Biological Sciences, Ohio University, Athens, OH 45701 USA

**Keywords:** warm acclimation, proteasome, metabolism, antioxidant, Antarctic fish

## Abstract

The Southern Ocean surrounding the Western Antarctic Peninsula region is rapidly warming. Survival of members of the dominant suborder of Antarctic fishes, the Notothenioidei, will likely require thermal plasticity and adaptive capacity in key traits delimiting thermal tolerance. Herein, we have assessed the thermal plasticity of several cellular and biochemical pathways, many of which are known to be associated with thermal tolerance in notothenioids, including mitochondrial function, activities of aerobic and anaerobic enzymes, antioxidant defences, protein ubiquitination and degradation in cardiac, oxidative skeletal muscles and gill of *Notothenia coriiceps* warm acclimated to 4°C for 22 days or 5°C for 42 days. Levels of triacylglycerol (TAG) were measured in liver and oxidative and glycolytic skeletal muscles, and glycogen in liver and glycolytic muscle to assess changes in energy stores. Metabolic pathways displayed minimal thermal plasticity, yet antioxidant defences were lower in heart and oxidative skeletal muscles of warm-acclimated animals compared with animals held at ambient temperature. Despite higher metabolic rates at elevated temperature, energy storage depots of TAG and glycogen increase in liver and remain unchanged in muscle with warm acclimation. Overall, our studies reveal that *N. coriiceps* displays thermal plasticity in some key traits that may contribute to their survival as the Southern Ocean continues to warm.

## Teaser

Notothenia coriiceps displays minimal remodeling of aerobic metabolic pathways in heart, oxidative skeletal muscle, or gill in response to warm acclimation, corresponding with a higher metabolic rate at elevated temperature. Nonetheless, antioxidant defenses are lower and levels of ubiquitinated proteins unchanged with warm acclimation.

## Introduction

The climate in Antarctica is rapidly changing, especially in the Western Antarctic Peninsula (WAP) region (Vaughan *et al.*, 2003; Turner *et al.*, 2014), where surface waters have increased by ~1°C during the past 50 years, and subsurface waters are projected to increase 0.4–0.6°C during the next century with an increase of as much as 1°C by the year 2200 (Meredith and King, 2005; Yin *et al.*, 2011). The sirens of warming are apparent in the collapse of ice shelves, sharp declines in Adélie penguin populations, shifts in phytoplankton distribution and a reduction in krill habitat and abundance (Clarke *et al.*, 2007; Stammerjohn *et al.*, 2008; Montes-Hugo *et al.*, 2009; Atkinson *et al.*, 2019). Much less is known about the impact of warming on the Antarctic fish fauna because of the inherent difficulties in monitoring fish populations in this remote, expansive and ice-laden region. Warming, coupled with other abiotic stressors, and challenges from anthropogenic influences (e.g. species’ invasions, commercial exploitation and contaminants) pose significant threats to Antarctic fishes and the integrity of the Antarctic ecosystem (Österblom and Sumaila, 2011; Hughes *et al.*, 2020; Webb *et al.*, 2020).

Members of the perciform suborder Notothenioidei dominate the fish fauna in the Southern Ocean, comprising 91% of the biomass of the benthic fish fauna on the continental shelf and 45% of the species (Eastman, 2005). Notothenioids are potentially vulnerable to warming because of their long evolution (12–22 MY) at temperatures less than 5°C, which has diminished their thermal plasticity compared with temperate fish species (e.g. Bilyk *et al.*, 2018). Moreover, 88% of notothenioids are endemic to the Southern Ocean (Eastman, 2005) and unlike fishes elsewhere, there is little to no opportunity for migration to more suitable habitats. Therefore, plasticity in key traits that influence thermal tolerance and fitness will likely be paramount to the survival of Antarctic fishes in a changing climate.

Encouragingly, cardiac performance, which seems to play a pivotal role in thermal tolerance for temperate fishes (ie Chen *et al.*, 2015; Ekstrom *et al.*, 2017; Jensen *et al.*, 2017) and Antarctic notothenioids (Joyce *et al.*, 2018b), improves with warm acclimation (Joyce *et al.*, 2018a). Both heart rate and cardiac output in warm-acclimated *Notothenia coriiceps* are maintained at temperatures above those of animals held at their habitat temperature (0°C) (Joyce *et al.*, 2018a), and in warm-acclimated *Pagothenia borchgrevinki*, the factorial scope of cardiac output is higher at temperatures between 4°C and 8°C compared with fishes held at ambient temperature (Franklin *et al.*, 2007).

The ability to sustain cardiac performance at elevated temperature is dependent, in part, on the production of ATP by aerobic metabolic pathways, and mitochondrial function, in particular, seems to play a central role in thermal tolerance in fishes (Iftikar *et al.*, 2015) This may be particularly true for Antarctic fishes that have a lower glycolytic capacity compared with temperate teleosts (Crockett and Sidell, 1990).

Despite improved cardiac performance following warm acclimation in *N. coriiceps*, it is of concern that routine metabolic rate ($ \dot{M}O_{2} $) remains elevated and is 1.4–2.0-fold higher in warm-acclimated animals compared with ambient ones, even after 6–9 weeks of acclimation (Egginton and Campbell, 2016, Joyce *et al.*, 2018a). The long-term energetic costs of maintaining higher metabolic rates may reduce the fitness of notothenioids by diverting energy away from key processes, such as reproduction and growth. It is unknown whether the lack of thermal compensation at the organismal level in *N. coriiceps* is due to a systemic inability to modulate the level of key metabolic enzymes, or if some tissues undergo metabolic remodelling while other tissues require a longer period of time to remodel and/or a greater deviation in temperature. Studies in both Antarctic notothenioids and temperate fishes have shown that metabolic remodelling varies among tissue types (Rodnick and Sidell, 1994; Rodnick and Sidell, 1997; Orczewska *et al.*, 2010; Jayasundara *et al.*, 2013).

Although rising temperatures will likely disrupt both top-down and bottom-up processes in the Southern Ocean food web (Constable *et al.*, 2014), there may be some physiological benefits to life in a warmer climate. Antarctic notothenioids have higher levels of molecular chaperones and ubiquitinated proteins and genes encoding antioxidants and proteins of the ubiquitin-proteasome pathway compared with temperate fishes, suggesting that cold temperature denatures proteins and that the higher oxygen solubility in cold waters may promote oxidative stress (Place *et al.*, 2004; Todgham *et al.*, 2007; Bilyk and Cheng, 2013; Kim *et al.*, 2019). Warmer temperatures may alleviate both of these stressors. Indeed, warm acclimation of notothenioids lowers oxidative stress (Bilyk and Cheng, 2014; Enzor and Place, 2014; Mueller *et al.*, 2014) that arises as a result of a mismatch between the production of reactive oxygen species (ROS) and antioxidants that eliminate ROS (Droge, 2002). When exposed to 4°C for 7 days, the icefish *Chionodraco rastrospinosus* and *N. coriiceps* have lower mRNA levels of the antioxidants superoxide dismutase (SOD) and/or catalase (CAT) in heart and oxidative skeletal muscle (Mueller *et al.*, 2014). Further, while initial exposure to 4°C increases levels of oxidized proteins (protein carbonyls) in gill and liver tissue of *Trematomus bernacchii* and *P. borchgrevinki*, carbonyl levels decline after 56 days of acclimation compared with animals held at ambient temperature (Enzor and Place, 2014). Additionally, polyubiquitin genes are down-regulated in the liver of the notothenioid *P. borchgrevinki* after exposure to 4°C for 4 days (Bilyk and Cheng, 2014). Together, the decline in levels of antioxidants and oxidized and ubiquitinated proteins in response to warm acclimation in Antarctic notothenioids may provide an energetic cost savings in a warmer climate, potentially minimizing the negative impacts of warming on potential disruptions in prey availability (Constable *et al.*, 2014) and feeding efficiency (Navarro *et al.*, 2019).

**Figure 1:**
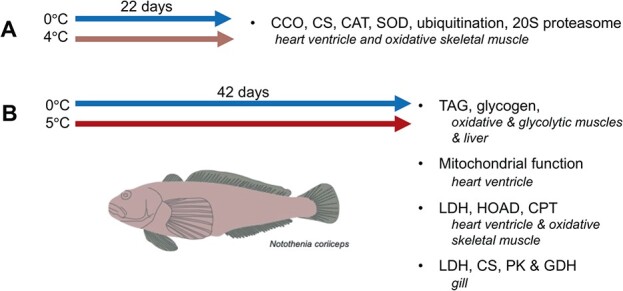
Experimental design used in acclimation experiments. In 2013 (A) *N. coriiceps* were acclimated to 4°C for 22 days. Tissues were harvested and activities of CS, CCO, CAT, SOD, levels of ubiquitinated proteins and the activity of the 20S proteasome were quantified in heart ventricle and oxidative skeletal muscle to determine the impacts of warm acclimation on metrics of oxidative stress. In 2017 (B) *N. coriiceps* were warm acclimated to 5°C for 42 days. Animals were harvested and levels of TAG and glycogen were measured in oxidative and glycolytic skeletal muscles and liver. Mitochondrial function was measured in mitochondria isolated from heart ventricles. And, a suite of metabolic enzymes (LDH, HOAD, CPT, PK, GDH and CS) were measured in oxidative skeletal muscle, heart ventricle and/or gill.

We sought to determine the thermal plasticity of physiological processes that might contribute to survival in a warming climate. The first set of acclimation experiments, conducted in 2013, was aimed to determine if warm acclimation alters oxidative stress by measuring the maximal activities of aerobic enzymes and antioxidants, as well as levels of ubiquitinated proteins and the activity of the 20S proteasome (Fig. 1A). The second set of acclimation experiments, conducted in 2017, was aimed to examine if improved cardiac performance in response to warm acclimation of *N. coriiceps* is associated with enhanced cardiac mitochondrial function and if the lack of thermal compensation in metabolic rate is reflected in the lack of metabolic remodelling at the tissue level (Fig. 1B). Together, these data provide insight to the thermal plasticity of key traits in *N. coriiceps* that might influence survival in a warming environment.

## Materials and methods

### Fish collection

*Notothenia coriiceps (Richardson 1844)* were collected from two locations in the WAP region [Low Island (63° 25′ S; 62° 10′ W) and Dallmann Bay (64° 10′ S; 62° 35′ W)] using benthic otter trawls and baited fish pots deployed from US ARSV *Laurence M. Gould* during the austral fall and winter of 2013*.* Fish were held in circulating seawater tanks onboard the ship before transfer to the aquarium at the US Antarctic Research Station, Palmer Station, where they were held in tanks with circulating seawater at 0.1 ± 0.5°C. In 2017, *N. coriiceps* were captured by hook and line off the pier at Palmer Station and then immediately transferred to the aquarium. All tanks were equipped with oxygen diffusers and blocks of frozen seawater were added as needed to maintain the temperature of the control tanks. Animals were fed a diet of chopped fish every other day and all uneaten food was removed from the tanks. Ammonia levels were measured every 2 days. All experimental procedures were approved by the University of Alaska IACUC committee (247598-11 and 570217-9).

### Warm acclimation

In 2013, *N. coriiceps* were placed in two 700 l insulated recirculating seawater tanks at 0.1 ± 0.5°C (three or four fish per tank). Fish were held for 24 hours before the temperature was increased 0.5°C per day for 6 days using 3-KW Elecro Titanium inline heaters (Aqualogic, San Diego, CA, USA) until the tanks reached 4°C. Flow rate in the heated tank was maintained at 1 gallon per minute (gpm) so that the volume of the tank turned over within 1 hour. Warm-acclimated animals were held at 4 ± 0.2°, while control animals were held in 2000 l tanks at 0.1 ± 0.5°C for 22 days (*n* = 7 for each temperature group).

In 2017, *N. coriiceps* were held in two 2000 l tanks (20–21 animals per tank), one of which was fitted with a 6-kW submersible heater (Aqualogic) to heat the tank as described above. A submersible pump was used to facilitate mixing. Animals were held at −0.6 ± 0.4° (control) or 5.1 ± 0.3°C (warm-acclimated) for 42 days. Flow rate was maintained between 5 (heated tank) and 8 (control tank) gpm so that the volume of the tank turned over within 2 hours. Sixteen animals from each temperature group were used for assays described herein.

The length of acclimation period was shorter and the acclimation temperature was lower in 2013 than 2017 because in 2013 we also attempted to warm-acclimate icefish, which have a lower thermal tolerance than *N. coriiceps* (Beers and Sidell, 2011).

Fish were euthanized with a sharp blow to the head followed by cervical transection. Hearts were excised and allowed to contract in notothenioid Ringer’s solution (260 mmol l^−1^ NaCl, 2.5 mmol l^−1^ MgCl_2_, 5.0 mmol l^−1^ KCl, 2.5 mmol l^−1^ NaHCO_3_, 5.0 mmol l^−1^ NaH_2_PO_4_, pH 8.0) to clear blood from the ventricular lumen. The atrium was removed, and the ventricle then blotted dry and weighed. The spleen was also removed, blotted dry and weighed. Heart ventricle, pectoral adductor muscle, glycolytic muscle, liver and gill tissues were flash frozen in liquid nitrogen and stored at −80°C to −70°C. Frozen tissues collected in 2013 were used for measuring the maximal activities of citrate synthase (CS), SOD and CAT, levels of ubiquitinated proteins and the activity of the 20S proteasome within 1 year. Fresh tissues were used for measuring the activity of cytochrome *c* oxidase (CCO) immediately upon harvest. Tissues collected in 2017 were used for measuring mitochondrial function, levels of triacylglycerol (TAG) and glycogen and maximal activities of CS (in gill only), 3-hydroxyacyl-CoA dehydrogenase (HOAD), carnitine palmitoyltransferase (CPT), lactate dehydrogenase (LDH), pyruvate kinase (PK) and glutamate dehydrogenase (GDH)—the latter two enzymes in gill tissue only. Enzymes were all assayed within 2 years of tissue collection; TAG and glycogen levels were measured in 2020. For all experiments, control and acclimated samples were measured at the same time.

### 20S proteasome activity

Activity of the 20S proteasome was measured based on the method developed by Shibatani and Ward (1995) and adapted for use on fish by Dobly *et al.* (2004). Frozen tissues were finely chopped on an ice-cold stage and then homogenized in five volumes of ice-cold lysis buffer (50 mM Tris–HCl, pH 8.0, 0.1 mM EDTA, 1.0 mM β-mercaptoethanol) using a Tissuemizer homogenizer (Tekmar) and Tenbroeck ground-glass tissue homogenizers. The homogenate was centrifuged at 20000 *g* for 1 hour at 4°C and the supernatant retained. Protein content of the supernatant was determined using a Bradford protein assay (Bradford, 1976) with bovine serum albumin (BSA) used for the standard curve.

Proteasome activity was measured using the proteasome-specific fluorogenic substrate LLVY-AMC (Enzo). The substrate was dissolved in DMSO (5.71 mM), then aliquoted and stored at −80°C until use. Although maximal activity of the 20S proteasome has been shown to require SDS (Shibatani and Ward, 1995), in our hands after testing in SDS concentrations ranging from 0% to 0.025%, maximal activity of the 20S proteasome was obtained in Tris buffer lacking SDS and so we omitted it from the reaction mixture. Activity was measured by incubating 50 μg protein from the supernatant with 40 μM LLVY-AMC in 22.5 μl 100 mM Tris–HCl (pH 8.0) for 60 minutes at 5°C. The reaction was determined to be linear for 90 minutes. The reaction was stopped by adding 225 μl 0.1 M sodium borate (pH 9.1) and 65 μl 1% SDS. Fluorescence of amido-4-methylcoumarin (AMC) was determined at excitation/emission wavelengths of 380 and 460 nm, respectively, on a Gemini EM Microplate Reader (Molecular Devices, Sunnyvale, CA, USA). Parallel samples were prepared by adding the proteasome inhibitor MG-132 (133 μM) (Enzo) prior to incubation. Activity was calculated by determining the concentration of AMC in the samples using the standard curve minus activity in the presence of MG-132. Activity was expressed as pmol AMC hr^−1^ 50 μg^−1^ protein. The standard curve was prepared using eight AMC concentrations between 3.3 μM and 44 μM. The samples and standard curve were measured in triplicate (*n* = 5–7).

### Levels of ubiquitinated proteins

Levels of ubiquitinated protein were measured using a method based on the study by Hofmann and Somero (1995) and modified by Todgham *et al.* (2007). Frozen tissues were finely chopped on an ice-cold stage and then homogenized in five volumes of ice-cold homogenization buffer (4% SDS [w/v], 1 mM EDTA, 50 mM Tris–HCl) supplemented with protease inhibitors (c*O*mplete Protease Inhibitor Cocktail Tablets, Roche, USA; pH 6.8) using a Tissuemizer homogenizer (Tekmar). Homegenization was completed using Tenbroeck ground-glass tissue homogenizers. Tissue homogenates were boiled 5 minutes to denature proteins. Homogenates were then centrifuged at 12000 *g* for 15 minutes at room temperature and the supernatant retained. Protein content of the supernatant was determined using a Bradford protein assay (1976) with BSA used for the standard curve. Supernatants were stored at −80°C.

Samples were diluted with Tris-buffered saline solution (TBS) (20 mM Tris–HCl, 140 mM NaCl, pH 7.6) to a concentration of 0.5 μg μl^−1^ for ventricle and pectoral adductor samples. A total of 1 μl of each sample was pipetted onto a 12 × 10 cm sheet of 0.2 μm nitrocellulose membrane (Amersham, GE Healthcare Life Sciences, Little Chalfont, UK) in triplicate. The protein was heat-fixed to the membrane at 65°C for 20 minutes. The membrane was then blocked with 5% nonfat milk powder dissolved in Tween-20 Tris-buffered saline solution (TTBS) (20 mM Tris–HCl, 140 mM NaCl, 0.01% Tween-20, pH 7.6, room temperature) for 1 hour. After blocking, the membranes were rinsed twice briefly with TTBS and then three times for 5 minutes each with TTBS. The membranes were incubated at 4°C with the ubiquitin conjugate primary antibody (mono-ubiquitinylated and polyubiquitinylated conjugates mAb produced in mice; Enzo Life Sciences, BML-PW8810, Farmdale, NY, USA), diluted 1:5000 in 5% nonfat milk powder dissolved in TTBS. Incubation times were 12.5 hours for ventricle and 15 hours for pectoral adductor. The membranes were then rinsed briefly twice with TTBS and then rinsed three times for 5 minutes each with TTBS. The membranes were incubated at room temperature with the secondary antibody (rabbit anti-Mouse IgG peroxidase antibody; A9044, Sigma-Aldrich, St. Louis, MO, USA), diluted 1:10000 in 5% nonfat dry milk powder in TTBS. Membranes with ventricle samples were incubated 1.5 hours and pectoral adductor samples for 2 hours. The membranes were then rinsed briefly twice with TTBS and then washed three times for 5 minutes each in TTBS and developed using a chemiluminescence kit (Amersham ECL Prime Western Blotting Detection Reagent, GE Healthcare), according to manufacturer’s specifications. Chemiluminescence was detected for 15 minutes using AlphaImager 3300 Imaging System (Protein Simple, San Jose, CA, USA). Samples from acclimated and control *N. coriiceps* were all loaded on the same membrane and absolute intensities quantified using ImageQuant TL software (GE Healthcare) to determine levels of ubiquitinated proteins. Measurements were made in triplicate with *n* = 5–7.

### Enzyme activities

Maximal activities of enzymes were measured using either a Perkin Elmer Lambda 25 or 40 spectrophotometer (Perkin-Elmer, Waltham, MA, USA) or a Beckman DU 640 (Beckman Coulter, Indianapolis, IN, USA) equipped with refrigerated, circulating water baths.

For CS assays, tissues were homogenized on ice (10% w/v) in 40 mM HEPES, 1 mM EDTA, 2 mM MgCl_2_, pH 7.8 at 1°C; for CPT, HOAD, PK and LDH assays, tissues were homogenized in 75 mM Tris–HCl, 1 mM EDTA, 2 mM MgCl_2_, pH 8.2 at 5°C using a Tekmar Tissumizer and completed with a Tenbroek ground glass homogenizer. This was followed by two 15-second sonication bursts with a Virtis Virsonic 100 Ultrasonic Cell Disrupter for HOAD and CPT assays. Tissue samples were stored frozen at −80°C following homogenization. Posterior gill filaments from each animal were placed on an ice-cold glass stage and the tissue gently scraped using a glass slide. Gill tissues were then homogenized (10% w/v) in 40 mM HEPES using a Tenbroeck ground glass homogenizer before being frozen at −70°C prior to assays.

### *Citrate synthase* (EC 2.3.3.1)

The maximal activity of CS was measured at 5°C ± 0.5°C in heart and pectoral muscle and at 2.5°C ± 0.5°C in the gill using a modification of the protocol described by Srere *et al.* (1963). The final reaction mixture contained 0.25 mmol l^−1^ 5,5′-dithiobis-2-nitrobenzoic acid (DTNB), 0.40 mmol l^−1^ acetyl coenzyme A (CoA), 0.5 mmol l^−1^ oxaloacetate, 75 mmol l^−1^ Tris–HCl and pH 8.2. Background activity was measured for 5 minutes in the absence of the initiating substrate oxaloacetate. The progress of the reaction was monitored by following the reduction of DTNB at 412 nm for 5 minutes after the addition of oxaloacetate. Activity is expressed as μmol product min^−1^ g^−1^ wet mass. Measurements were made in triplicate with *n* = 6–7.

### *Cytochrome c oxidase* (EC 1.9.3.1)

The maximal activity of CCO was measured at 5°C ± 0.5°C as described by Wharton and Tzagoloff (1967). Tissue was homogenized in 50 mmol l^**−**1^ K_2_HPO_4_/KH_2_PO_4_, 0.05% Triton X-100 and pH 7.5. The assay medium consisted of 10 mmol l**-**1 K_2_HPO_4_/KH_2_PO_4_, 0.65% (w/v) reduced (Fe2+) cytochrome c and 0.93 mmol l^**−**1^ K_3_Fe(CN)_6_. Maximal activities were measured by following the oxidation of reduced cytochrome c at 550 nm. Measurements were made in triplicate with *n* = 6–7.

### *Catalase* (EC 1.11.1.6)

Maximal activity of CAT was quantified at 5 ± 0.5°C by monitoring the decomposition of hydrogen peroxide at 240 nm as described by Beers Jr and Sizer (1952). Briefly, tissues were homogenized in nine volumes (v/w) of 50 mmol l^−1^ phosphate buffer, pH 7.8. Background rates were determined for 2 minutes by monitoring rates of hydrogen peroxide decomposition in a reaction mixture containing 10 or 25 μl tissue homogenate in 50 mmol l^−1^ phosphate buffer and pH 7.8 in a final volume of 1 ml. Enzyme reactions were initiated by adding a final concentration of 11 mmol l^−1^ hydrogen peroxide to the reaction mixture. Activity of CAT was expressed as μmol min^−1^ g wet tissue^−1^. Measurements were made in triplicate with *n* = 6.

### *Superoxide dismutase* (EC 1.15.1.1)

Maximal activity of SOD was quantified at 5 ± 0.5°C by monitoring the reduction of cytochrome *c* at 550 nm (McCord and Fridovich, 1969; Crapo *et al.*, 1978). Briefly, tissues were homogenized in nine volumes (v/w) of ice-cold 50 mmol l^−1^ potassium phosphate, 0.1 mmol l^−1^ EDTA, pH 7.8. The reduction of 0.01 mmol l^−1^ acetylated cytochrome *c* was measured in the presence of 0.05 mmol l^−1^ xanthine, 0.01 mmol l^−1^ KCN and xanthine oxidase (XO). The final concentration of XO was determined each day to obtain a reduction rate of cytochrome *c* of 0.02 OD min^−1^. One unit of SOD activity is defined as the amount of SOD needed to achieve 50% inhibition of the reduction rate of cytochrome *c*. Homogenates were diluted until a reduction rate of 0.01 ± 0.008 OD min^−1^ was achieved. Activity of SOD was expressed as Units g wet tissue^−1^. Measurements were made in triplicate with *n* = 6.

### *3-hydroxyacyl-CoA dehydrogenase* (EC 1.1.1.35)

Maximal activity of HOAD was measured at 2.5°C using a procedure described by Hansen and Sidell (1983) and following the reduction of NAD^+^ at 340 nm. The final reaction mixture contained 50 mM imidazole, 1 mM EDTA, 1 mM KCN, 0.15 mM NAD^+^ and 0.1 mM acetoacetyl-CoA, adjusted to a pH of 7.7 at 1°C. Background activity was monitored in the absence of acetoacetyl-CoA for five minutes. The reaction was initiated with addition of acetoacetyl-CoA and activity measured for five minutes. Measurements were carried out in triplicate with *n* = 8.

### *Lactate dehydrogenase* (EC 1.1.1.27)

The maximal activity of LDH was measured at 2.5°C ± 0.5°C using a modification of the method described by Hansen and Sidell (1983). The final reaction mixture contained 50 mmol l^−1^ imidazole, 1 mmol l^−1^ KCN, 0.15 mmol l^−1^ NADH and 2.5 mmol l^−1^ pyruvate, adjusted to pH 7.7 at 2.5°C. The oxidation of NADH was monitored at 340 nm for 3 minutes in the absence of pyruvate. The reaction was then initiated by addition of pyruvate and the change in absorbance at 340 nm monitored for 3 minutes. All measurements were made in triplicate with *n* = 7 for ventricle tissue and *n* = 6 for pectoral muscle tissue and gill.

### *Carnitine palmitoyltransferase* (EC 2.3.1.21)

Maximal activity of CPT was measured at 2.5°C ± 0.5°C. The final reaction mixture contained 40 mM HEPES, 1.5 mM EDTA, 0.25 mM 5,5′-dithiobis (2-nitrobenzoic acid) (DTNB), 1.25 mM carnitine, 0.035 mM palmitoleoyl-CoA, adjusted to pH of 8.2 at 1°C. The reduction of DTNB was monitored at 412 nm in the absence of carnitine for 3 minutes. The reaction was then initiated by addition of carnitine and the change in absorbance at 412 nm was monitored for at least 10 minutes. Measurements were carried out in triplicate with *n* = 8.

### *Pyruvate kinase* (2.7.1.40)

Maximal activity of PK was measured at 2.5°C ± 0.5°C in gill tissues by a slight modification of the method described by Bergmeyer (1974) and Hansen and Sidell (1983). Reaction medium consisted of 50 mM imidazole, 150 mM KCl, 10 mM MgSO_4_, 0.15 mM NADH, 5 mM adenosine 5ʹ- diphosphate (ADP), 10 U/ml LDH and 2.5 mM phosphoenolpyruvate (PEP) (pH = 7.0 at 2.5°C). The oxidation of NADH was followed at 340 nm for 6 minutes in the absence (control) and presence of PEP. All assays were run in triplicate with *n* = 6.

### *Glutamate dehydrogenase* (EC 1.4.1.2)

GDH was assayed in gill 2.5°C ± 0.5°C only using the method described previously by Chamberlin *et al.* (1991). The final reaction mixture contained 50 mM imidazole, 250 mM ammonium acetate, 0.1 mM EDTA, 1 mM ADP, 0.1 mM NADH and 14 mM α-ketoglutarate (pH = 8.3 at 2.5°C), the latter of which was used to initiate the reaction. Reactions were monitored at 340 to follow the oxidation of NADH in the absence/presence of the substrate α-ketoglutarate for 5 minutes. Measurements were made in triplicate with *n* = 6.

### Levels of TAG and glycogen

Levels of TAG and glycogen were measured using assay kits and following the manufacturer’s protocols (Sigma-Aldrich Life Science, MAK266 for TAG and MAK016 for glycogen). Tissues were homogenized in nine volumes of 5% Nonidet P 40 for measuring TAGs and in PBS for measuring glycogen, using a Tissumizer (Tekmar, Cincinnati, OH, USA). For TAGs, homogenates were heated at 95°C for 2–5 minutes twice, and for glycogen measurements, homogenates were heated at 95°C for 8 minutes and then centrifuged at 13000 *g* for 5 minutes. Samples were stored at −80°C until use. Absorbance was measured at 570 nm using a SpectraMax Plus384 plate reader (Molecular Devices, Sunnyvale, CA, USA) and final concentrations were determined using a standard curve. All samples were measured in duplicate for TAG and triplicate for glycogen (*n* = 6).

### Mitochondrial function

Heart ventricles from two *N. coriiceps* were pooled for each mitochondrial preparation (*n* = 5). Ventricles were minced into 1–2-mm-sized blocks on an ice-cold stage and then gently homogenized with six passes of a pestle in a Tenbroeck homogenizer in eight volumes of isolation buffer (0.1 M sucrose, 140 mM KCl, 10 mM EDTA, 5 mM MgCl_2_, 20 mM HEPES, 0.5% fatty-acid free BSA, pH 7.3 at 4°C, 435 mOsm). The homogenate was then centrifuged at 1400 *g* for 5 minutes at 4°C. The supernatant was collected and centrifuged at 9000 *g* for 10 minutes at 4°C. The resulting pellet was gently resuspended in 1 ml of isolation buffer and then diluted to 10 ml with isolation buffer and centrifuged at 1400 *g* for 5 minutes at 4°C. The supernatant was then decanted and centrifuged at 11000 *g* for 10 minutes at 4°C. The mitochondrial pellet was gently resuspended in assay buffer (173 mM sucrose, 135 mM KCl, 5 mM KH_2_PO_4_, 20 mM HEPES, 0.5% BSA, pH 7.3 at 4°C, 435 mOsm). Protein concentration was determined using the Bradford Assay (Bradford, 1976).

Mitochondrial respiration rates were measured at 5°C and 15°C in duplicate (when possible) using an SI130 microcathode oxygen electrode with a SI782 dual channel meter and RC300 respiration cell (all from Strathkelvin, North Lanarkshire, Scotland) cooled with a circulating water bath. Mitochondrial function was measured at 5°C because it is difficult to measure mitochondrial function at temperatures below 5°C using the Strathkelvin system and 5°C was the acclimation temperature of *N. coriiceps*. Mitochondrial function was measured at 15°C because it approximates the upper thermal tolerance limit (critical thermal maximum, CT_MAX_) of *N. coriiceps* (Beers and Sidell, 2011). Electrodes were calibrated daily at each temperature. Respiration rates were measured in 1 ml of assay buffer with 31–73 μl of mitochondrial protein for measurements at 5°C (average of 480 μg) and 17–40 μl for measurements at 15°C (average of 262 μg). All rates were monitored for a minimum of 3 minutes. The Krebs cycle was initiated by addition of 1 mM malate and 5 mM pyruvate to measure state 2 respiration rates, 0.5 mM of ADP was then added to measure state 3 respiration rates. Following depletion of ADP, state 4 respiration rates were monitored. A second aliquot of ADP was then added to measure states 3 and 4 rates a second time and the two measurements were averaged. A total of 1 μg m^−1^ of oligomycin was added to inhibit ATP synthase, followed by 10 μM FCCP to measure maximal rate of respiration (ETS). Then 12.5 μM of rotenone, 15 mM malonate, 10 μM antimycin were added to inhibit complexes I, II and III, respectively. Then 0.5 mM TMPD and 2 mM ascorbate were added to measure the activity of CCO. All biochemicals were purchased from Sigma-Aldrich (St. Louis, MO, USA) for these assays and measurements of enzyme activities (described above).

**Table 1:** Physical characteristics of ambient and warm-acclimated *N. coriiceps*

Temperature	Body mass (g)	Heart mass (g)	RVM	Spleen mass (g)	Spleen: body mass	K
Acclimated 22 days						
0° C (*n* = 7) 4**°**C (*n* = 7)	1505 ± 76 1079 ± 64^*^	1.46 ± 0.08 (*n* = 6) 1.05 ± 0.09^*^	0.10 ± 0.002 (*n* = 5) 0.10 ± 0.005	5.79 ± 1.22 4.51 ± 0.73	0.38 ± 0.07 0.42 ± 0.06	1.50 ± 0.12 (*n* = 6) 1.73 ± 0.04^*^
Acclimated 42 days						
0° C (*n* = 16) 5**°**C (*n* = 16)	583 ± 38 598 ± 37	0.56 ± 0.03 0.60 ± 0.04	0.10 ± 0.003 0.10 ± 0.004	1.80 ± 0.16 2.39 ± 0.21^**^	0.32 ± 0.03 0.39 ± 0.02^**^	1.73 ± 0.04 1.69 ± 0.02

Data are means ± S.E.M.; RVM: relative ventricular mass (100*ventricular mass/body mass); K: condition factor (100* body mass/length^3^).

^*^Significant difference (*P* ˂ 0.05) between 22-day ambient and warm-acclimated groups.

^**^Significant difference (*P* ˂ 0.05) between 42-day ambient and warm-acclimated groups.

### Statistical analyses

A Grubb’s Test was used to identify outliers, which were removed. Normality was determined using a Shapiro–Wilk test. Measurements of maximal activity of HOAD in hearts of control animals were not normally distributed and transforming the data did not achieve a normal distribution. However, the data were normally distributed based on the D’Agostino & Pearson test and so were included in the study. A Principal Component Analysis (PCA) was used to qualitatively evaluate differences between acclimation groups within each tissue in metrics of oxidative stress and activities of metabolic enzymes (from 2013). Pearson product–moment correlation coefficients were used to evaluate relationships between antioxidants (SOD, CAT activity), pro-oxidants (CS and CCO activity) and oxidative damage (proteasome activity and ubiquitin levels) within heart and pectoral adductor muscle. A two-way ANOVA followed by a Sidak’s multiple comparisons test was used to determine significant differences between acclimation groups in mitochondrial function (with assay and acclimation temperatures as main factors) and in metabolic enzyme activities, proteasome activity and levels of protein ubiquitination in heart ventricle and pectoral adductor muscle (with tissue and acclimation temperature as the main factors). A Student’s *t*-test was used to detect significant differences in physical characteristics and enzyme activities in the gill between temperature groups, and a Mann–Whitney test was used to identify significant differences in glycogen and TAG levels between temperature groups within each tissue. Prism 9.0 (GraphPad Software, San Diego, CA, USA) was used for all statistical analyses with significance set at *P* < 0.05.

## Results

There were no mortalities among the warm-acclimated fishes in either 2013 or 2017. In 2017, one ambient animal died during the acclimation period.

### Physical characteristics

The body mass of *N. coriiceps* acclimated to 4°C for 22 days in 2013 was significantly lower than that of control animals (1079 ± 64, 1505 ± 76 g, respectively; Table 1; *P* < 0.05). Despite their smaller mass, the condition factor of the warm-acclimated animals was significantly higher than that of ambient animals (Table 1; *P* < 0.05). Given the relatively short acclimation period, it is unlikely that the differences in body mass were the result of warm acclimation. Heart mass was also smaller in the warm-acclimated fishes (*P* < 0.05) but relative ventricular mass did not differ between the two groups (*P* > 0.05; Table 1). There were no significant changes in any other measured physical characteristics as a result of warm acclimation for 22 days (Table 1; *P* > 0.05).

There were no differences in body mass or condition factor between control animals and ones acclimated to 5°C for 42 days in 2017 (Table 1; *P* > 0.05). However, the spleen mass of *N. coriiceps* acclimated to 5°C for 42 days was 1.3-fold higher than that of animals held at ambient temperature, resulting in a 1.2-fold greater spleen: body mass ratio (Table 1; *P* < 0.05) and suggesting that warm acclimation may increase red blood cell stores.

### Effects of 22 days of warm acclimation on aerobic metabolic enzymes, antioxidant activity, proteasome activity and protein ubiquitination

**Figure 2:**
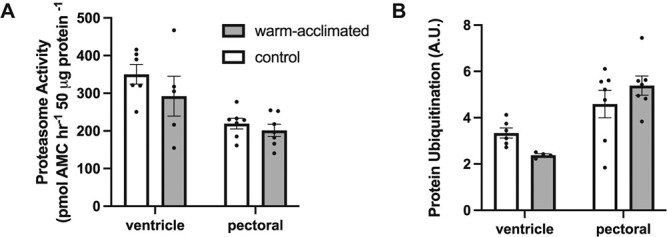
Maximal activity of the 20S proteasome measured at 5°C (A) and levels of ubiquitinated protein (B) in *N. coriiceps* acclimated to 4°C for 22 days. Data are means ± S.E.M. All data points are shown and each circle represents an individual (*n* = 4–7).

PCA revealed that in both heart ventricles and oxidative skeletal muscle, ambient and warm-acclimated animals tended to differ in metrics of oxidative capacity and stress (CS, CCO, SOD, CAT, proteasome activity, ubiquitinated proteins) (Supplementary Fig. S1). PC1 explained 43% and PC2 explained 32% of the variation among individuals in heart ventricles (Supplementary Fig. S1A); in pectoral adductor muscle, PC1 explained 41% of the variation and PC2 explained 26% of the variation among individuals (Supplementary Fig. S1B). In general, antioxidant levels were significantly lower in warm-acclimated *N. coriiceps* compared with ambient animals (SOD: *F* = 6.548, *P* = 0.019; CAT: *F* = 35.68, *P* < 0.0001). The maximal activity of CAT was lower in hearts and oxidative skeletal muscle, and SOD activity was lower in oxidative skeletal muscle of warm-acclimated *N. coriiceps* compared with control animals (Table 2; *P* < 0.05 for all comparisons). Despite a decrease in antioxidant capacity with warm acclimation, the activity of CCO tended to be higher in warm-acclimated animals in pectoral muscle and heart ventricle (*F* = 3.287, *P* = 0.084). Consistent with this, regression analysis showed a significant inverse correlation between CCO and SOD activity in the ventricle (*P* = 0.011; Supplementary Fig. S2). In pectoral muscle there was a significant inverse correlation between SOD activity and ubiquitin levels (*P* = 0.049) and there tended to be an inverse correlation between CS activity and ubiquitin levels in the heart (*P* = 0.057). There were no significant differences in levels of ubiquitinated proteins or the activity of the proteasome in warm-acclimated versus control animals (Fig. 2*; P* > 0.05) but there was a significant interaction between temperature and tissue in levels of ubiquitinated proteins (*F* = 4.753, *P* = 0.016).

**Table 2:** Maximal activity of metabolic enzymes in ambient and warm-acclimated *N. coriiceps*

Enzyme	Acclimation temperature	Heart ventricle (μmol product min^−1^ g wet mass ^−1^)	Oxidative skeletal muscle (μmol product min^−1^ g wet mass ^−1^)
Acclimated 22 days (measured at 5°C)			
CS (*n* = 6–7)	0° C4° C	19.98 ± 0.9320.42 ± 0.98	44.58 ± 2.3244.22 ± 2.72
CCO (*n* = 6–7)	0° C4° C	30.96 ± 2.9635.83 ± 1.33	45.44 ± 3.7251.55 ± 3.63
CAT (*n* = 6)	0°C4°C	444.76 ± 12.74367.93 ± 17.00^*^	378.29 ± 11.30285.6 ± 15.04^*^
SOD (*n* = 6)	0°C4° C	2154.35 ± 98.892013.94 ± 62.05	3815.03 ± 284.652998.20 ± 217.79^*^
Acclimated 42 days (measured at 2.5°C)			
HOAD (*n* = 8)	0° C5°C	3.08 ± 0.202.88 ± 0.36	4.43 ± 0.404.71 ± 0.46
LDH (*n* = 6–7)	0°C5° C	141.22 ± 12.54134.04 ± 16.91	49.65 ± 3.4959.21 ± 6.48
CPT (*n* = 8)	0°C5°C	0.18 ± 0.010.17 ± 0.02	0.39 ± 0.020.39 ± 0.02

Data are means ± S.E.M.

^*^Significant difference between ambient and warm-acclimated fishes (*P* < 0.05).

### Effects of 42 days of warm acclimation on metabolism

**Figure 3:**
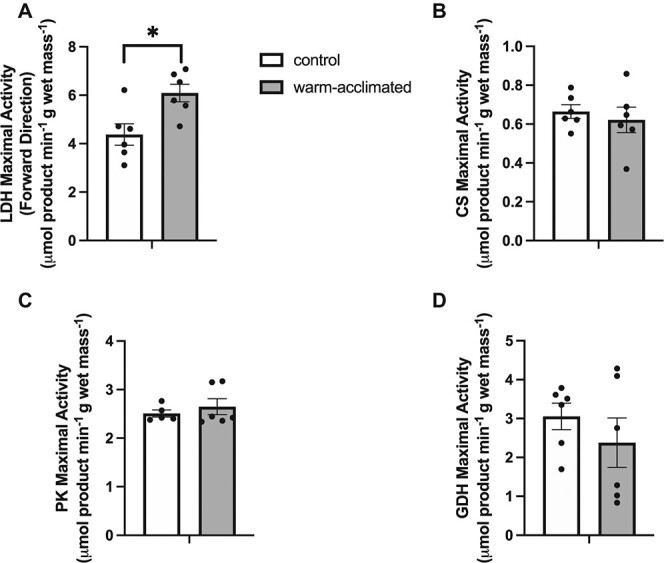
Maximal activities of metabolic enzymes in gill in *N. coriiceps* acclimated to 5°C for 42 days and at measured at 2.5°C. Data are means ± S.E.M. All data points are shown with each circle representing an individual. ^*^Significant differences (*P* ˂ 0.05) between control and warm-acclimated groups. *n* = 5–6.

The maximal activity of enzymes involved in both aerobic and anaerobic pathways did not change in response to warm acclimation of *N. coriiceps*, even after 42 days, in most tissues sampled (Table 2; Fig. 3). An exception was in gill, where there was a significant increase in the maximal activity of LDH in response to warm acclimation (Fig. 3A; *P* < 0.05). Consistent with this, PCA did not reveal distinct clustering of metabolic enzyme activity for ambient and warm-acclimated animals in any of the tissues (data not shown).

The maximal activities of metabolic enzymes were assayed at a common temperature. Thus, *in vivo* enzyme activity would be higher in warm-acclimated compared with ambient animals, consistent with their higher *M*O_2_ (Egginton and Campbell, 2016; Joyce *et al.*, 2018a). Despite higher metabolic rates at elevated temperature, levels of both TAGs and glycogen in the liver were significantly higher in warm-acclimated *N. coriiceps* compared with ambient animals (Table 3; *P* < 0.05) but unchanged in muscle (Table 3; *P* > 0.05).

**Table 3:** Levels of TAG and glycogen in tissues of ambient and warm-acclimated *N. coriiceps*

Metabolite	Acclimation temperature	Liver	Glycolytic skeletal muscle	Oxidative skeletal muscle
Acclimated 42 days				
TAG (μmol g wet mass^−1^)	0°C 5°C	56.9 ± 1.9 70.6 ± 5.1^*^	0.7 ± 0.2 0.5 ± 0.1	2.9 ± 0.4 2.7 ± 0.6
Glycogen (mg wet mass ^−1^)	0°C 5°C	19.2 ± 6.5 45.6 ± 0.8^*^	2.9 ± 0.2 2.8 ± 0.3	

Data are means ± S.E.M.; *n* = 5–6.

^*^Significant difference between ambient and warm-acclimated fishes (*P* < 0.05).

### Effects of warm acclimation on mitochondrial function

**Figure 4:**
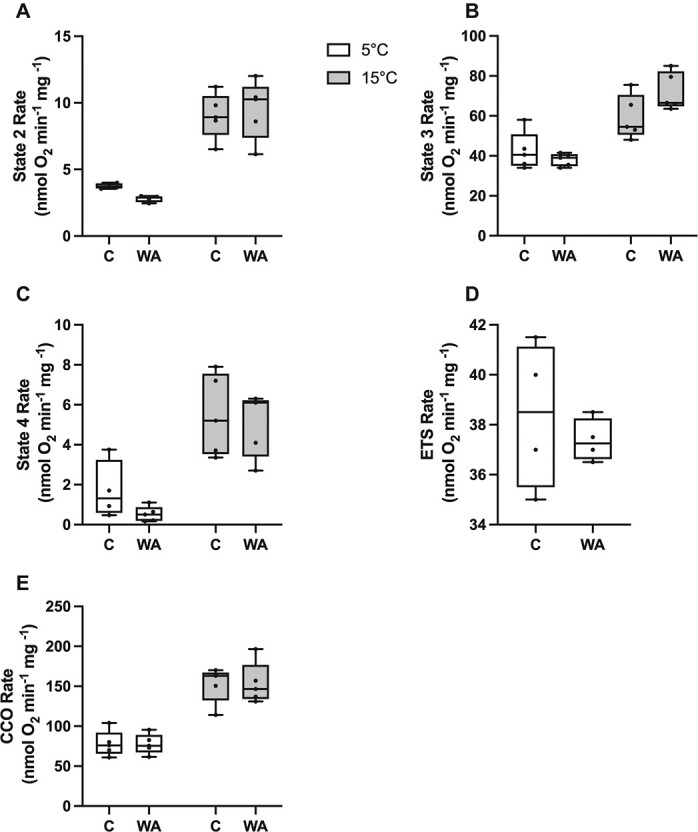
Mitochondrial function in mitochondria isolated from heart ventricle of *N. coriiceps* acclimated 5°C for 42 days and measured at 5°C or 15°C. All data points are shown with each circle representing an individual. C, control; WA, warm-acclimated. *n* = 4–5.

There were no significant differences in state 2 rate, state 3 rate, maximal rate of respiration (ETS) or activity of CCO between mitochondria isolated from cardiac muscle of warm-acclimated and ambient *N. coriiceps* when measured at 5°C or 15°C (Fig. 4; *P* > 0.05); although there was a significant interaction between acclimation and assay temperature in state 3 rates of respiration (*F* = 4.676, *P* = 0.046). All rates of respiration were higher when measured at 15°C than at 5°C (Fig. 4; *P* < 0.05).

## Discussion

Previous studies have shown that rates of oxygen consumption ($ \dot{M}O_{2} $) are not thermally compensated for in *N. coriiceps* in response to warm acclimation, with rates 1.4–2.0-fold higher in warm-acclimated animals at 5°C compared with animals held at ambient temperature (Egginton and Campbell, 2016; Joyce *et al.*, 2018a). Consistent with this, we find only modest metabolic remodelling at the cellular level with activities of anaerobic and aerobic metabolic enzymes in gill and oxidative skeletal and cardiac muscles mostly similar between warm-acclimated and ambient animals after 42 days of acclimation to 5°C when assayed at a common temperature. Somewhat surprising, though, the activities of enzymatic antioxidants (SOD and CAT) were lower in *N. coriiceps* acclimated to 4°C for only 22 days compared with ambient animals, and the activity of the proteasome and levels of ubiquitinated proteins were unchanged. These results suggest that although metabolic rates are elevated at warmer temperatures in *N. coriiceps*, there does not appear to be an increased cost of protein degradation to remove oxidatively damaged proteins.

### Warm acclimation has little effect on capacities for energy metabolism

We detected no change in the maximal activities of enzymes involved in aerobic metabolism (CS, CCO), or fatty acid oxidation (HOAD, CPT) or anaerobic metabolism (LDH) in heart or oxidative muscle of *N. coriiceps* in response to warm acclimation to 5°C for 42 days or 4°C for 22 days (Table 2). Some studies of Antarctic fishes from both the WAP (Windisch *et al.*, 2011) and the higher latitude region of McMurdo Sound (Seebacher *et al.*, 2005; Davis *et al.*, 2018) have found evidence of thermal metabolic plasticity; although it is not always clear if the response is beneficial. For example, in hearts of *T. bernacchii*, the activities of CS, LDH and HOAD decline following warm acclimation to 4.5°C for 14 days but cardiac performance does not improve (Jayasundara *et al.*, 2013). The activity of CS increased in liver and gill of *T. bernacchii* following acclimation to 4°C for 4 weeks, but the activity of LDH remained unchanged (Enzor *et al.*, 2017). However, in juvenile *T. bernacchii*, acclimation to 2°C for 4 weeks resulted in a decrease in CS activity in skeletal muscle, coinciding with a decline in $ \dot{M}O_{2} $, suggesting greater metabolic plasticity in juveniles (Davis *et al.*, 2018). Overall, metabolic thermal plasticity varies widely among Antarctic fish species, tissue types and life stage (Lannig *et al.*, 2005; Seebacher *et al.*, 2005; Windisch *et al.*, 2011; Jayasundara *et al.*, 2013; Enzor *et al.*, 2017; Davis *et al.*, 2018).

Few studies have investigated metabolic remodelling in the gills of Antarctic notothenioids in response to warm acclimation. Studies in temperate fishes, however, indicate that the gill possesses a high degree of morphological and biochemical thermal plasticity (e.g. Nilsson *et al.*, 2012; Gibbons *et al.*, 2018). For example, within 5 days of acclimation to an elevated temperature, crucian carp and goldfish show significant reductions in the amount of interlamellar cell mass, thereby increasing surface area of the respiratory (lamellae) surface (Sollid and Nilsson, 2006). Membrane remodelling also occurs following temperature acclimation in both Antarctic and temperate teleosts (Robertson and Hazel, 1995; Biederman *et al.*, 2021), as well as adjustments in metabolism in response to a variety of stressors (Arjona *et al.*, 2009; Srivastava *et al.*, 2017; Syahputra *et al.*, 2020).

Prior studies have shown that the activities of gill Na^+^/K^+^-ATPase increase with warm acclimation in two species of Antarctic notothenioids by between 1.3-fold and 1.9-fold (Gonzalez-Cabrera *et al.*, 1995) and 1.4-fold in *N. coriiceps* (Biederman *et al.*, 2021), likely requiring an increase in ATP production. While we did not observe any changes with acclimation in CS activity, there was a 1.4-fold increase in the activity of LDH with warm acclimation, indicating that the rise in ATP demand by the gill may be met by the glycolytic pathway.

### Warm acclimation has little impact on mitochondrial function

When measured at their acclimation temperature, cardiac output is 2.7-fold higher in warm-acclimated *N. coriiceps* compared with animals at 0°C but $ \dot{M}O_{2} $ increases only 1.4-fold, resulting in a significantly lower arterio-venous extraction in warm-acclimated animals and suggesting lower O_2_ demand at the tissue level (Joyce *et al.*, 2018a). Associated with improved cardiac output with warm acclimation, the temperature at which the heart fails increases from 15.0°C to 17.7°C (Joyce *et al.*, 2018a). Together, these data suggest mitochondria may become more efficient following warm acclimation, lowering the amount of O_2_ required to synthesize ATP and improving cardiac performance at elevated temperature. While there were no significant differences in cardiac mitochondrial function between warm-acclimated and ambient *N. coriiceps*, the trends in our data suggest that mitochondrial function at elevated temperature may improve with warm acclimation. State 4 rates, indicative of proton leak, tend to be lower in warm-acclimated animals (Fig. 4C), and state 3 rates tend to be higher, especially when measured at 15°C. The net effect of changes in states 3 and 4 respiration rates is a trend towards an increase in the respiratory control ratio following warm acclimation (*F* = 3.561, *P* = 0.079), indicative of a decrease in proton leakage. When measured at 5°C, only 1.4% of oxygen consumed in state 3 contributes to proton leak in warm-acclimated animals compared with more than twice that (3.5%) in ambient animals, and when measured at 15°C, 7.0% of oxygen consumed in state 3 contributes to proton leak in warm-acclimated animals compared with 9.3% in ambient ones. Similarly, warm acclimation of common intertidal triplefin fish, *Forsterygion lapillum*, to 24°C results in lower rates of mitochondrial proton leak in skeletal muscle compared with animals at 18°C (Khan *et al.*, 2014), although this trend is not consistent across all fish species (e.g.; Iftikar *et al.*, 2015). Interestingly, when exposed to an acute increase in temperature (CT_MAX_), state 4 rates increase relative to state 3 rates in cardiac mitochondria of *N. coriiceps*, which may contribute to the decline in ATP levels and cardiac failure at CT_MAX_ (O'Brien *et al.*, 2018). The trend towards a decrease in mitochondrial proton leakage at higher temperatures following warm acclimation may be attributable to mitochondrial membrane remodelling or changes in the surface area of the inner mitochondrial membrane, both of which have been shown to influence leak in mammalian mitochondria (Porter *et al.*, 1996). While we have not investigated mitochondrial architecture, our previous work has shown that the proportion of long chain fatty acids is higher in cardiac mitochondrial membranes from warm-acclimated *N. coriiceps*, corresponding with a lower fluidity compared with ambient animals when measured at a common temperature (Biederman *et al.*, 2021), which may, in fact, contribute to a decrease in proton leakage.

Mitochondrial metabolic thermal plasticity in Antarctic notothenioids, in general, seems low compared with temperate fish species. For example, in the congeneric species, *Notothenia rossii*, acclimation to 7°C for 4–5 weeks does not significantly alter state 3 respiration rates, proton leak or mitochondrial membrane composition in liver (Strobel *et al.*, 2013). It is possible that mitochondrial remodelling is tissue specific. Yet, in the temperate killifish, *Fundulus heteroclitus*, mitochondrial metabolism is more plastic in brain and liver mitochondria compared with cardiac mitochondria (Chung and Schulte, 2015; Chung *et al.*, 2017).

### Warm acclimation lowers antioxidant defences

Given that the activity of aerobic metabolic enzymes did not change with warm acclimation, one might anticipate a similar lack of change or even an increase in levels of antioxidant defences to combat a potential increase in ROS production with a higher metabolic rate, and yet warm acclimation seems to induce a compensatory decrease in levels of oxidized macromolecules and antioxidants in some tissues of notothenioids (Enzor and Place, 2014). The majority of ROS are produced by mitochondria during oxidative phosphorylation when unpaired electrons react with oxygen, forming superoxide ions (Boveris *et al.*, 1972). Superoxide is detoxified by SOD, producing peroxide (another ROS) that is then reduced to water by a suite of enzymatic antioxidants, including CAT and peroxidases (Kausar *et al.*, 2018). An imbalance between the production of ROS and antioxidant defences results in oxidative damage to proteins, DNA and lipids. An acute increase in temperature increases oxidative stress in notothenioids with increases in levels of oxidized proteins and lipids and antioxidant genes (Thorne *et al.*, 2010; Mueller *et al.*, 2012; Klein *et al.*, 2017). Yet we find that warm acclimation of *N. coriiceps* to 4°C for only 22 days leads to a decrease in maximal activities of the antioxidant CAT in cardiac and oxidative skeletal muscle and SOD in oxidative skeletal muscle, suggesting that rates of ROS production decline in these tissues despite a lack in change in the activity of the aerobic metabolic enzymes CS and CCO when assayed at a common temperature. Consistent with this, there is no change in levels of ubiquitinated proteins in response to warm acclimation.

Alterations in mitochondrial membrane composition have been shown to affect ROS production in mammals (Saborido *et al.*, 2011; Vial *et al.*, 2011), and thus remodelling of mitochondrial membrane composition with warm acclimation observed in *N. coriiceps* hearts (Biederman *et al.*, 2021) may minimize ROS production at elevated temperature. Moreover, in the absence of thermal compensation of state 3 respiration rates, the higher flux through the ETS in warm-acclimated animals may enhance the turnover rate of ATP, thereby lowering membrane potential and rates of ROS production (Munro and Treberg, 2017). In support of this, in Atlantic salmon (*Salmo salar*) rates of oxygen consumption of cardiac mitochondria are higher in warm-acclimated (20°C) salmon compared with 12°C-acclimated salmon when measured across a range of temperatures, yet rates of ROS production decline by 10–40% (Gerber *et al.*, 2020).

Oxidized and thermally denatured proteins are degraded by the 26S and 20S proteasome (Shringarpure and Davies, 2002). Whereas degradation by the 26S proteasome requires proteins to be polyubiquitinated, degradation by the 20S proteasome does not (Davies, 2001; Shringarpure *et al.*, 2003). Previous studies have shown that levels of ubiquitinated proteins in gill, heart, liver and spleen and the activity of the 20S proteasome in gill and liver are higher in Antarctic fishes compared with most related cold-temperate species, suggesting that protein damage either caused by cold temperature or oxidative stress and/or protein misfolding is higher at cold temperature and may be alleviated in a warmer environment (Todgham *et al.*, 2007, 2017). Consistent with this, transcriptome analysis has shown that mRNA levels of genes encoding some subunits of the proteasome (such as proteasome subunit beta type-7) are highest in the liver of the Antarctic eelpout (*Pachycara brachycephalum*) at −1° to 0°C, and remain constant at 3° to 5°C, but are down-regulated when acclimated to temperatures of 7°C and higher (Windisch *et al.*, 2014), and mRNA levels of polyubiquitin-B are down-regulated in liver in *P. borchgrevinki* after 4 days at 4°C (Bilyk and Cheng, 2014). Similarly, we find that levels of ubiquitinated proteins tend to decline in response to warm acclimation to 4°C for 22 days in heart ventricles (*P <* 0.05 for *t*-test) and there was no significant change in proteasome activity with warm acclimation.

### Energy stores are maintained with warm acclimation

Despite a higher metabolic rate at 5°C than at 0°C (Joyce *et al.*, 2018a), the condition factor of *N. coriiceps* was unaffected by warm acclimation at 5°C for 42 days (Table 1) and TAG and glycogen levels increased in liver and were unchanged in muscle. This is in contrast to the congeneric species, *N. rossii*, in which resting metabolic rate did not decline in response to warm acclimation to 7°C but the hepatosomatic index was 50% lower than that of animals at 1°C after 29–26 days of acclimation (Strobel *et al.*, 2012). In *T. bernacchii*, despite thermal compensation of routine metabolic rate in response to warm acclimation to 4°C, growth rate declined by 84%, likely due to lower assimilation rates (Sandersfeld *et al.*, 2015) and lipid liver content tended to be lower in warm-acclimated *T. bernacchii* (Sandersfeld *et al.*, 2015). For the 42-day acclimation studies in which we measured glycogen and TAG stores, we used *N. coriiceps* captured by hook and line in near-shore, shallow areas that are likely more thermally variable than deeper off shore areas and the animals were smaller and likely younger than ones captured off shore. These animals may be more resilient to warming than older, more deep dwelling notothenioids. In support of this, juvenile *T. bernacchii* have a greater capacity to acclimate than adults with $ \dot{M}O_{2} $ of juveniles declining after only 4 weeks at elevated temperature (Davis *et al.*, 2018). Taken together, these data suggest that climate warming may alter the energy budget of some, but not all, Antarctic notothenioids. The higher metabolic rates of notothenioids at elevated temperature may reflect a new steady state condition that is not necessarily deleterious (Todgham and Mandic, 2020).

## Conclusions

Our data suggest some, albeit limited, capacity for metabolic remodelling in response to warm acclimation in *N. coriiceps* with a tendency towards lower rates of proton leakage in cardiac mitochondria and a lower requirement for antioxidant defences in hearts and oxidative skeletal muscle, and an increase in glycolytic metabolism in the gill to support higher rates of ion transport and osmoregulation (i.e. Na+/K + -ATPase activity; Biederman *et al.*, 2021). Coupled with improved cardiac performance at elevated temperature (Joyce *et al.*, 2018a) and membrane remodelling (Biederman *et al.*, 2021), our results indicate that despite more than 12 MY of evolution in a cold, stable environment, *N. coriiceps* has retained some degree of thermal plasticity. Whether plasticity exists in the key traits that will influence persistence in a changing climate is not entirely clear. However, mitochondrial function and membrane leakage in *N. coriiceps* and the haemoglobinless icefish, *Chaenocephalus aceratus*, are associated with differences in thermal tolerance, suggesting that plasticity in these traits may contribute to improved fitness (O'Brien *et al.*, 2018; Evans *et al.*, 2021). Studies of adaptive capacity in these traits would improve predictions of the vulnerability of notothenioids to climate change.

Thermal plasticity varies considerably among notothenioids in differing habitats (e.g. Bilyk *et al.*, 2018), and given that many species of notothenioids have a circumpolar distribution (Gon and Heemstra, 1990), it is unknown whether thermal plasticity varies among populations, with perhaps high-latitude populations more vulnerable to warming than those inhabiting more thermally variable lower latitudes, as has been shown for different species (Bilyk and Devries, 2011). Additional complicating factors are that thermal plasticity varies with life stage (Dahlke *et al.*, 2020) and warming is only one of several abiotic stressors intensifying with climate change along with ocean acidification and the frequency and severity of hypoxic events (Deutsch *et al.*, 2015; Flynn *et al.*, 2015; Davis *et al.*, 2018; Todgham and Mandic, 2020). Further studies on the impacts of multiple stressors are warranted and advised by the Scientific Committee on Antarctic Research (Chown *et al.*, 2022).

Notothenioids are one node within a complex marine food web system in the Southern Ocean with disruptions in one node rippling throughout the ecosystem. Ultimately the persistence of notothenioids in a changing environment will be determined not only by thermal plasticity and adaptive capacity in key traits at critical life stages, but also by prey availability, disease susceptibility and competition from invasive species. In addition to rapid warming in the WAP, also of great concern is the increasing krill harvest that is disrupting higher trophic levels (Kruger, 2019) and human activities, introducing invasive species (Hughes *et al.*, 2020) and environmental contaminants (Krasnobaev *et al.*, 2020; Lacerda *et al.*, 2020; Webb *et al.*, 2020). Our results presented here and the findings of others (Egginton and Campbell, 2016, Joyce *et al.*, 2018a) show that notothenioids display minimal metabolic plasticity with warming, indicating that the integrity of the Antarctic ecosystem with sufficient prey availability to support higher metabolic rates is essential for surviving in a changing climate. Sustaining an intact Antarctic ecosystem will require expansion of marine protected areas, which are under the purview of the Commission for the Conservation of Antarctic Marine Living Resources, established in 1982 to sustainably manage fisheries in the Southern Ocean, and governed by consensus among its 25 member States and the European Union. Studies such as ours, aimed at understanding the capacity of Antarctic fishes to endure climate change, are most effective when used to inform policy and establish protective measures (Cooke *et al.*, 2020); in the absence of which, we are merely documenting the biology of another species at risk for extinction.

## Data availability

Data are available in Dryad https://doi.org/10.5061/dryad.37pvmcvp8.

## Supplementary Material

suppl_coac054
